# Relationship Between Salivary Metabolites and Skeletal Muscle Index in Older Male Patients: A Retrospective Observational Pilot Study to Identify Potential Biomarkers

**DOI:** 10.1155/jare/5554198

**Published:** 2026-01-11

**Authors:** Tatsuya Hayasaka, Shigeo Ishikawa, Ayuka Narisawa, Machika Moriya, Hiroaki Toyama, Masahiro Sugimoto

**Affiliations:** ^1^ Department of Anesthesiology, Yamagata University Hospital, Yamagata, Japan; ^2^ Department of Dentistry, Oral and Maxillofacial-Plastic and Reconstructive Surgery Faculty of Medicine, Yamagata University, Yamagata, Japan, yamagata-u.ac.jp; ^3^ Institute for Advanced Biosciences, Keio University, Tsuruoka, Japan, keio.ac.jp; ^4^ Institute of Medical Science, Tokyo Medical University, Tokyo, Japan, tokyo-med.ac.jp

**Keywords:** nutritional assessment, perioperative management, salivary metabolomics, sarcopenia, skeletal muscle index

## Abstract

**Background:**

Nutritional assessment during the perioperative period is critical, particularly for older patients at high risk for complications. Bioelectrical impedance analysis (e.g., InBody) is commonly used to assess skeletal muscle mass via the skeletal muscle index (SMI). However, its use is limited in patients with contraindications, including implantable cardiac devices.

**Objective:**

Therefore, this study explored a noninvasive alternative: assessing the relationship between salivary metabolites and SMI to identify potential biomarkers associated with muscle mass.

**Methods:**

This retrospective study analyzed data from male preoperative patients who had both InBody analysis and salivary metabolomics data collected between January 2021 and March 2024. Salivary metabolites, including butyrate and hexanoate, were quantified using capillary electrophoresis time‐of‐flight mass spectrometry (CE‐TOF MS). Body composition parameters, including SMI, and blood nutritional indicators, were obtained. Volcano plot analysis identified metabolites significantly differing between patients with SMI ≥ 7.0 and < 7.0 kg/m^2^. Receiver operating characteristic (ROC) curve analysis evaluated the discriminatory ability of individual variables. Univariate and multivariate logistic regression analyses identified factors associated with SMI status.

**Results:**

Patients with low SMI were significantly older than those with high SMI. While body composition indices differed significantly, general nutritional blood markers remained comparable. Volcano plot analysis showed significantly higher salivary butyrate and hexanoate levels in patients with SMI ≥ 7.0 kg/m^2^ compared to those with SMI < 7.0 kg/m^2^. ROC curve analysis demonstrated significant discriminatory ability for butyrate, hexanoate, and age. Univariate analysis identified age as significantly associated with SMI status based on odds ratio. Multivariate analysis using stepwise variable selection retained age (OR: 1.155, *p*‐value = 0.070) and hexanoate (OR: 0.980, *p*‐value = 0.269) in the final model.

**Conclusions:**

This exploratory study suggests that salivary metabolites, particularly butyrate and hexanoate, along with age, may serve as potential indicators for discriminating SMI status. These findings suggest the potential utility of salivary metabolites as noninvasive biomarkers for assessing muscle mass in the perioperative setting. This could enable early sarcopenia detection and enhanced nutritional management in older patients, particularly those with contraindications.

**Trial Registration:** UMIN Clinical Trials Registry: UMIN000057185

## 1. Introduction

Nutritional assessment during the perioperative period is one of the factors that significantly impacts patient prognosis. Older patients, particularly those with frailty, are at a higher risk of postoperative complications, experiencing a higher incidence of acute muscle weakness and worsening frailty, especially as intensive care unit‐acquired weakness (ICU‐AW). Therefore, assessment and subsequent management of nutritional status is essential [1–6]. Sarcopenia, defined as the progressive decline in skeletal muscle mass and function associated with aging, is recognized as a contributing factor to the risk of perioperative complications, necessitating its assessment [7–11]. Recent research has found a significant association between sarcopenia prevalence in the older population and systemic inflammation markers, highlighting the role of chronic inflammation in its pathophysiology [12].

The InBody (InBody Co., Ltd, Korea), a device that uses bioelectrical impedance analysis (BIA), has been widely used to analyze body composition. BIA is used to calculate skeletal muscle index (SMI), which plays an important role in sarcopenia diagnosis [13–16]. The Asian Working Group for Sarcopenia guidelines have set the SMI cutoff at 7.0 kg/m^2^ for men; patients with a value below this are diagnosed with sarcopenia [17]. However, the use of InBody is prohibited for patients using implantable cardiac devices, life‐support medical devices, or similar equipment due to the potential side‐effects of weak electric currents. Using BIA devices such as InBody to assess the nutritional status of these patients is thus challenging, warranting alternative noninvasive assessment methods.

In recent years, metabolomic approaches have been used to identify biomarkers related to sarcopenia and muscle mass. Several studies have identified serum metabolites that correlate with muscle mass and function, providing valuable insights into sarcopenia pathophysiology. For example, recent research has demonstrated associations between specific blood metabolites, such as hypoxanthine, galactose, and mannose, with sarcopenia status [18]. These findings suggest that metabolic profiles could serve as useful biomarkers for sarcopenia diagnosis and monitoring. However, while blood–based metabolomic analyses offer valuable information, they still require invasive sampling, which may not be ideal for frequent monitoring during the perioperative period.

Various methods, including blood tests and imaging diagnosis, are used to assess nutritional status; however, saliva has recently attracted attention as a noninvasive biological sample that can be continuously monitored with minimal patient burden. Saliva contains various metabolic substances, such as amino and organic acids, and analyzing its composition could allow for the assessment of the whole body’s metabolic state [19, 20]. Given the success of metabolomic approaches in identifying sarcopenia biomarkers in blood, extending this methodology to salivary samples represents a logical next step toward developing truly noninvasive assessment tools.

Despite these advances in metabolomic research, knowledge regarding the relationship between metabolites in saliva and the metabolic dynamics of skeletal muscle, particularly in relation to sarcopenia and SMI, is insufficient. Exploring the associations between salivary metabolites and body composition, particularly skeletal muscle mass as measured by SMI, could provide insights into potential noninvasive biomarkers for nutritional assessment in the perioperative setting.

The rationale for investigating salivary metabolites as biomarkers for muscle mass assessment is that saliva can be collected noninvasively, allowing for repeated sampling with minimal patient burden compared to blood tests. Previous research has also demonstrated that salivary metabolites can reflect systemic metabolic changes, as many blood‐borne metabolites are transferred to saliva through passive diffusion and active transport mechanisms [21]. For example, the muscle protein breakdown biomarker 3‐methylhistidine has been detected in saliva [22]. A past study of ours found that salivary 3‐methylhistidine was a significant prognostic factor for overall survival in patients with oral squamous cell carcinoma, suggesting that saliva can reflect systemic conditions [23]. These collective findings support the potential of salivary metabolomics as a method for assessing muscle mass status in clinical settings.

If certain salivary metabolites prove to be significantly associated with skeletal muscle mass, it would enable noninvasive, appropriate nutritional assessments for a larger number of perioperative patients. Therefore, this study aimed to explore the relationship between salivary metabolites and skeletal muscle mass as measured by SMI in older male patients, with a particular focus on identifying specific metabolites that may be associated with muscle mass status.

## 2. Materials and Methods

### 2.1. Study Design and Participants

**Figure 1:**
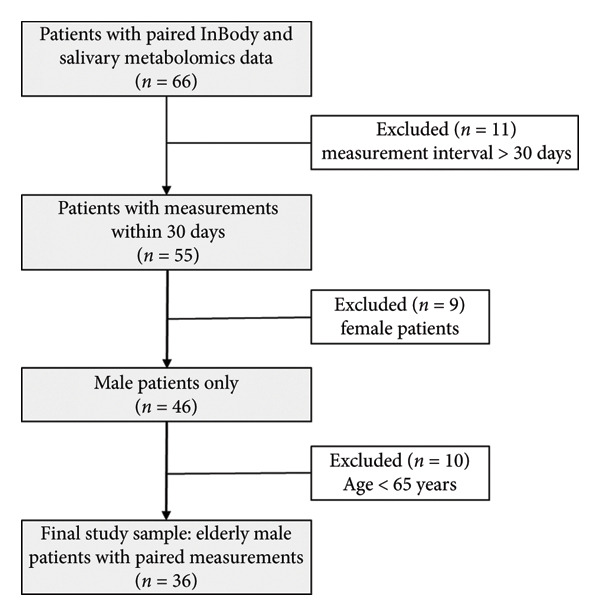
Flowchart of participant selection process. The study began with 66 patients identified from our institutional database who had both InBody bioelectrical impedance analysis and salivary metabolomics data between January 2021 and March 2024. Patients were excluded if the interval between measurements exceeded 30 days (11 excluded, leaving 55 patients). To ensure homogeneity, the analysis was restricted to male patients, excluding 9 females (46 patients remaining). Finally, focusing on the elderly population, patients younger than 65 years were excluded (10 excluded). The final analytical cohort consisted of 36 elderly male patients with paired body composition and salivary metabolite measurements.

This retrospective observational study was conducted from January 1, 2021, to March 31, 2024. We analyzed data from male patients aged 65 or older with both body composition analysis (InBody) from the Anesthesiology preoperative outpatient department and salivary metabolomics data from dental visits. Of the initial 66 patients with both measurements, we included only those with saliva and InBody measurements performed within 30 days of each other (*n* = 55). After restricting the analysis to male patients (*n* = 46) and those aged 65 years or older, the final analytical cohort consisted of 36 patients. The detailed participant selection process is illustrated in Figure 1. We included only male patients in this study to account for sex differences in skeletal muscle mass. Salivary metabolomics analysis has been routinely performed at Yamagata University Hospital as part of oral examinations, as demonstrated in previous studies [23–26]. This established protocol enabled the collection and analysis of saliva samples for metabolomic profiling.

### 2.2. Data Collection

Patient characteristics (including age, sex, height, and weight) and clinical laboratory data (such as lymphocyte count, serum albumin level, and C‐reactive protein [CRP]) were retrieved from the medical records. Based on our institutional reference range, CRP was categorized either as normal (< 0.14 mg/dL) or elevated (≥ 0.14 mg/dL). The following body composition–related indices were obtained from the InBody measurements: body water content, protein content, mineral content, body fat content, body mass index, body fat percentage, muscle mass by body region, fat mass by body region, water content by body region, intracellular and extracellular water content, skeletal muscle mass, basal metabolic rate, body cell mass, SMI, and phase angle.

### 2.3. Saliva Collection and Sample Preparation

**Figure 2:**
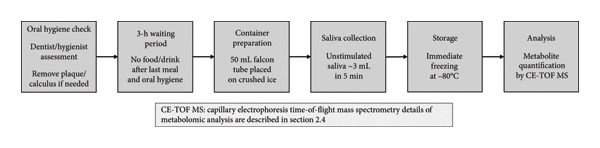
Flowchart of saliva sample collection protocol. The saliva collection procedure consisted of six sequential steps. Following oral hygiene assessment by a dentist or dental hygienist (with plaque/calculus removal if necessary), participants underwent a 3‐h waiting period with no food or drink after their last meal and oral hygiene procedures. Saliva collection was performed using 50‐mL Falcon tubes placed on crushed ice. Approximately 3 mL of unstimulated saliva was collected over 5 min, immediately frozen at −80°C, and subsequently analyzed for metabolite quantification using CE‐TOF MS.

The protocol for saliva collection has been previously described [23, 24, 27] (Figure 2). A dentist or dental hygienist confirmed the oral hygiene of all participants before saliva collection. If remarkable dental plaque and calculus deposits were present, they were removed using a toothbrush without dentifrice or ultrasonic scaling. Saliva collection was performed at least 3 h after the last food intake and oral hygiene procedures. Saliva samples were collected in 50‐mL Falcon tubes placed in a paper cup filled with crushed ice. Approximately 3 mL of unstimulated saliva was obtained over 5 min without any stimulation, and the samples were immediately stored at −80°C after collection.

### 2.4. Metabolomic Analysis

Metabolomic analyses of the saliva samples were performed using an Agilent G1969A LC/MSD time‐of‐flight (TOF) system, exactly as previously described [28]. For quality control, all metabolites were quantified using absolute quantification with standard mixtures, eliminating the need for batch‐to‐batch correction using QC samples that is typically required in relative quantification methods. First, frozen saliva was thawed and dissolved at room temperature. To remove macromolecules, the samples were centrifuged through a 5‐kDa cut‐off filter (Millipore) at 9100 × g for at least 2.5 h at 4°C. Next, 45 μL of the filtrate was transferred to a 1.5‐mL Eppendorf tube, to which 5 μL of water containing 2 mM methionine sulfone, 2‐(N‐morpholino) ethane sulfonic acid, d‐camphor‐10‐sulfonic acid, sodium salt, 3‐aminopyrrolidine, and trimesate was added and mixed. Charged metabolites in positive and negative modes were quantified using capillary electrophoresis time‐of‐flight mass spectrometry. The raw data were processed using MasterHands software [29]. Metabolites were identified by matching the corresponding m/z values and migration times, and absolute concentrations were determined by comparing the peak areas (normalized to those of the internal standards) with those of the standard mixtures.

### 2.5. Statistical Analysis

For this study, patients were categorized into two groups based on their SMI: high SMI (≥ 7.0 kg/m^2^) and low SMI (< 7.0 kg/m^2^), in accordance with the Asian Working Group for Sarcopenia guidelines for diagnosing sarcopenia in Asian men [17]. For between‐group comparison, Fisher’s exact and Mann–Whitney *U* tests were used for categorical and continuous variables, respectively.

Continuous variables are presented as median (interquartile range), and categorical variables are presented as frequency (percentage). To detect inflammation, CRP was analyzed as a categorical variable (normal: < 0.14 mg/dL; elevated: ≥ 0.14 mg/dL) based on our institutional reference range. Fisher’s exact test was applied to between‐group comparisons. A volcano plot was used to identify significantly different salivary metabolites between patients with high and low SMI. Specifically, a volcano plot analysis was performed using a fold‐change cutoff of 2.0 and a false discovery rate (FDR) of *p*‐value < 0.05. Principal component analysis (PCA) was also performed to provide an overview of the distribution of metabolites in patients with high and low SMI. The area under the curve (AUC) of the receiver operating characteristic (ROC) curve was calculated to evaluate the discriminatory ability of each individual variable for predicting SMI status, with the focus on metabolites identified from the volcano plot analysis and other relevant variables, including age. For variables with significant discriminatory ability (AUC with *p*‐value < 0.05), individual ROC curves were generated and sensitivity and specificity were calculated. Subsequently, to identify factors associated with low SMI status, we performed univariate logistic regression analyses using the variables that showed significant discriminatory ability in the ROC analysis, and other potentially relevant indicators. The odds ratio (OR) with 95% confidence intervals (CI) was calculated for each variable. Finally, we conducted multivariate logistic regression analysis using stepwise variable selection with the forward method based on the Akaike information criterion (AIC). Variables with a *p*‐value < 0.25 in the univariate analysis or those considered clinically relevant were included as candidates for the stepwise selection process, following the purposeful selection method [30].

All statistical analyses were performed using R version 4.4.3 (R Foundation for Statistical Computing, Vienna, Austria) and MetaboAnalyst 6.0 (http://www.metaboanalyst.ca/), and *p*‐value < 0.05 was considered statistically significant.

### 2.6. Ethics Approval

The Ethical Review Committee of Yamagata University Faculty of Medicine approved this study’s protocol (approval number: 2024‐60). This retrospective observational study used existing clinical data. As this was a retrospective analysis of previously collected samples and data, the study was retrospectively registered with UMIN on March 2, 2025, to ensure transparency and adherence to publication standards. Claude (Anthropic, San Francisco, CA, USA), a large language model, was used to improve English grammar and readability of the manuscript. It was not used for data entry, data analysis, interpretation of results, or formulation of research conclusions.

## 3. Results

Table 1 presents the patient characteristics, and Table 2 shows the comparison of nutritional status indicators between the low‐ and high‐SMI groups. Age significantly differed between the two groups, with the low‐ and high‐SMI groups having a median [interquartile range] age of 77 [73.5–80] and 72 [69.0–77] years, respectively (*p*‐value = 0.042). Regarding body composition, significant differences were found in the parameters obtained from InBody between the two groups. However, no significant differences were observed in general nutritional indicators such as serum albumin levels, lymphocyte counts, and CRP between the groups.

**Table 1:** Baseline characteristics of 36 male patients undergoing preoperative assessment.

Characteristics	Median [IQR] or *n* (%)
Age (years)	72.5 [69.0–77.3]

Sex (%)	Male: 36 (100.0)

ASA physical status	II: 26 (72.2), III: 10 (27.8)

Surgical procedure	Gastric: 17 (47.2)
	Pancreatic: 12 (33.3)
	Esophageal: 2 (5.6)
	Hepatic: 2 (5.6)
	Others: 3 (8.3)

Height (cm)	165.5 [160.4–169.9]

Weight (kg)	64.6 [58.7–72.8]

BMI (kg/m^2^)	23.8 [21.4–26.9]

Skeletal muscle mass (kg)	26.1 [23.7–28.8]

Skeletal muscle index (kg/m^2^)	7.1 [6.8–7.8]

Body fat mass (kg)	17.0 [12.8–22.3]

Total body water (kg)	35.1 [32.4–39.0]

Protein mass (kg)	9.3 [8.6–10.2]

Mineral mass (kg)	3.2 [2.9–3.5]

Basal metabolic rate (kcal)	1397 [1313–1504]

Phase angle (°)	4.9 [4.5–5.4]

Serum albumin (g/dL)	4.0 [3.8–4.1]

Lymphocyte count (× 10^3^/μL)	1.5 [1.0–1.9]

C‐reactive protein (mg/dL)	Normal (< 0.14 mg/dL): 19 (52.8), Elevated (≥ 0.14 mg/dL): 17 (47.2)

*Note:* Values are presented as median [interquartile range] or *n* (%).

Abbreviations: ASA, American Society of Anesthesiologist; BMI, body mass index; SMI, skeletal muscle index.

**Table 2:** Comparison of body composition parameters and nutritional indicators between low‐SMI (< 7.0 kg/m^2^) and high‐SMI (≥ 7.0 kg/m^2^) groups.

Characteristics	Low SMI (*n* = 11)	High SMI (*n* = 25)	*p*‐value
Age (years)	77 [73.5–80]	72 [69.0–77]	0.042^∗^
ASA physical status^‡^	II: 6 (54.5), III: 5 (45.5)	II: 20 (80.0), III: 5 (20.0)	0.224
Height (cm)	159.0 [157.1–164.7]	166.9 [162.2–170.2]	0.023^∗^
Weight (kg)	60.9 [54.95–64.55]	68.2 [59.50–76.10]	0.010^∗^
BMI (kg/m^2^)	23.3 [20.55–24.55]	24.3 [22.70–27.60]	0.064
Skeletal muscle mass (kg)	23.0 [21.85–24.25]	28.0 [25.70–30.90]	< 0.001^∗^
Skeletal muscle index (kg/m^2^)	6.5 [6.35–6.7]	7.5 [7.10–8.0]	< 0.001^∗^
Body fat mass (kg)	16.0 [12.5–19.4]	17.4 [13.40–23.7]	0.731
Total body water (kg)	31.7 [30.1–33.1]	37.8 [34.7–41.0]	< 0.001^∗^
Protein mass (kg)	8.3 [7.9–8.8]	9.9 [9.1–10.9]	< 0.001^∗^
Mineral mass (kg)	2.81 [2.795–3.03]	3.32 [3.090–3.63]	0.001^∗^
Basal metabolic rate (kcal)	1294 [1251–1341]	1473 [1384–1568]	< 0.001^∗^
Phase angle (°)	4.5 [4.45–4.7]	5.1 [4.60–5.6]	0.009^∗^
Serum albumin (g/dL)	3.9 [3.4–4.1]	4.0 [3.8–4.2]	0.359
Lymphocyte count (× 10^3^/μL)	1.49 [0.93–1.745]	1.65 [1.29–1.91]	0.354
C‐reactive protein (mg/dL)^‡^	Normal: 6 (54.5), Elevated: 5 (45.5)	Normal: 13 (52.0), Elevated: 12 (48.0)	1.000

*Note:* Values are presented as median [interquartile range] or *n* (%).

Abbreviations: ASA, American Society of Anesthesiologist; BMI, body mass index; SMI, skeletal muscle index.

^‡^Fisher’s exact test was used for categorical variables; Mann–Whitney *U* test was used for continuous variables.

^∗^ *p*‐value < 0.05.

**Figure 3:**
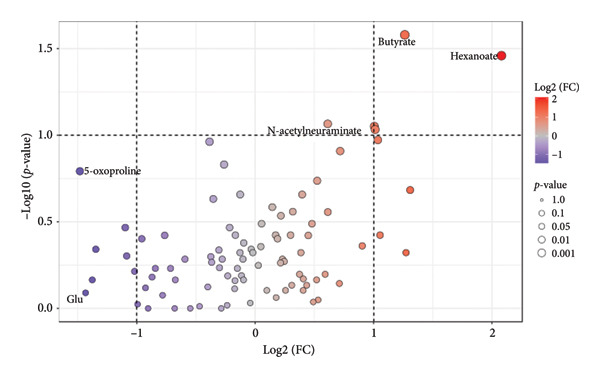
Volcano plot of salivary metabolites by skeletal muscle index (SMI) status. Differences in salivary metabolite levels between the groups with SMI ≥ 7.0 and SMI < 7.0 kg/m^2^. Data were normalized using log transformation and autoscaling. Two metabolites, hexanoate and butyrate, showed significant differences with a fold‐change (FC) threshold of 2.0 and a false discovery rate of *p*‐value < 0.05.

Figure 3 shows volcano plots of the saliva samples between the high‐ and low‐SMI groups. As shown in Figure 3 and Table 3, the volcano plot analysis identified two metabolites (butyrate and hexanoate) that were significantly different between the high‐ and low‐SMI groups (*p*‐value < 0.05). These metabolites showed fold changes of 2.401 and 4.229, respectively, indicating substantially higher levels in patients with SMI ≥ 7.0 kg/m^2^ compared to those with SMI < 7.0 kg/m^2^. Two additional metabolites, 1,3‐diaminopropane and 6‐phosphogluconate, showed similar fold changes but did not reach statistical significance (*p*‐value = 0.089 and *p*‐value = 0.093, respectively).

**Table 3:** Salivary metabolites showing significant differences between high‐ and low‐SMI groups identified by volcano plot analysis.

	FC	log2 (FC)	*p*‐value
Butyrate	2.401	1.264	0.026^∗^
Hexanoate	4.229	2.080	0.035^∗^
1,3‐Diaminopropane	2.009	1.006	0.089
6‐Phosphogluconate	2.018	1.013	0.093

*Note:* log2 (FC), Log2 transformed Fold Change values.

Abbreviations: FC, fold change; SMI, skeletal muscle index.

^∗^ *p*‐value < 0.05.

**Figure 4:**
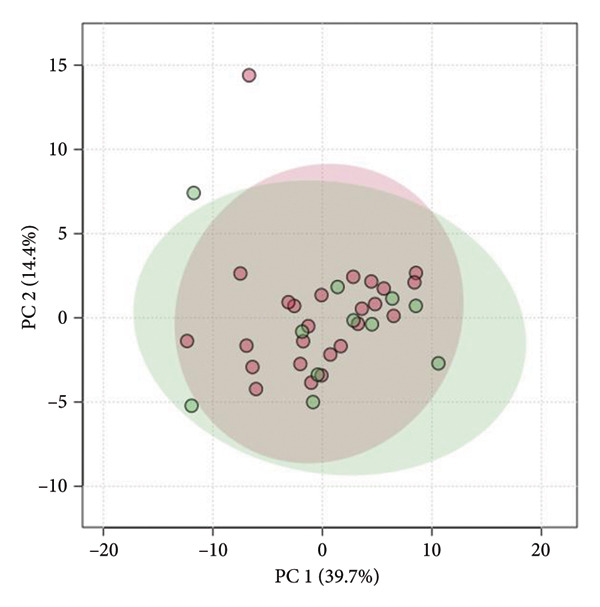
Principal component analysis (PCA) of salivary metabolite profiles. This score plot represents the results of the PCA based on salivary metabolite profiles. Green and red dots indicate samples from patients with SMI < 7.0 and ≥ 7.0 kg/m^2^. The contribution rates for PC1 and PC2 are 39.7% and 14.4%, respectively. SMI, skeletal muscle index; PCA, principal component analysis; PC, principal component.

Figure 4 shows the score plots of PCA after log transformation. The green and red plots indicate the samples obtained from the low‐ and high‐SMI groups, respectively. Additionally, the first principal component (PC1) and second principal component (PC2) contributed 39.7% and 14.4% to the variance, respectively.

We performed ROC curve analysis to evaluate the discriminatory ability of individual variables for SMI status. Three variables demonstrated significant AUC values: butyrate (AUC: 0.735, 95% CI: 0.547–0.922, *p*‐value = 0.014), hexanoate (AUC: 0.724, 95% CI: 0.516–0.931, *p*‐value = 0.035), and age (AUC: 0.716, 95% CI: 0.529–0.903, *p*‐value = 0.023). Figure 5 shows the ROC curves for these three variables. Butyrate exhibited the highest discriminatory ability (AUC: 0.735), with an optimal cut‐off of 206.3 μmol/L (sensitivity: 0.818, specificity: 0.640). Hexanoate also showed good discriminatory ability (AUC: 0.724), with an optimal cut‐off of 6.5 μmol/L (sensitivity: 0.636, specificity: 0.880). Age also demonstrated significant discriminatory ability (AUC: 0.716), with an optimal cut‐off of 74.5 years (sensitivity: 0.727, specificity: 0.720). Detailed cut‐off values and performance metrics are provided in Supporting Table. Traditional nutritional indicators, including serum albumin (AUC: 0.598, *p*‐value = 0.365), CRP (AUC: 0.560, *p*‐value = 0.556), and lymphocyte count (AUC: 0.600, *p*‐value = 0.358) did not show significant discriminatory ability for SMI status.

Figure 5Receiver operating characteristic (ROC) curves for variables with significant discriminatory ability for skeletal muscle index (SMI) status. (a) ROC curve for salivary butyrate in discriminating SMI status. (b) ROC curve for salivary hexanoate in discriminating SMI status. (c) ROC curve for age in discriminating SMI status. This figure displays the ROC curves for the three variables with significant discriminatory ability for SMI status. (a) Butyrate showed the highest AUC (0.735, 95% CI: 0.547–0.922, *p*‐value = 0.014) with sensitivity of 0.818 and specificity of 0.640. (b) Hexanoate demonstrated good discriminatory ability (AUC: 0.724, 95% CI: 0.516–0.931, *p*‐value = 0.035) with sensitivity of 0.636 and specificity of 0.880. (c) Age showed significant discriminatory ability (AUC: 0.716, 95% CI: 0.529–0.903, *p*‐value = 0.023) with sensitivity of 0.727 and specificity of 0.720. ROC, receiver operating characteristic; SMI, skeletal muscle index.

**Figure:**
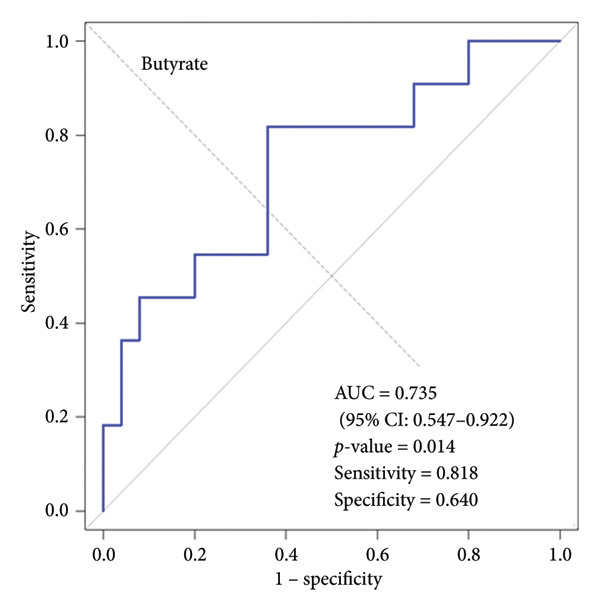
(a)

**Figure:**
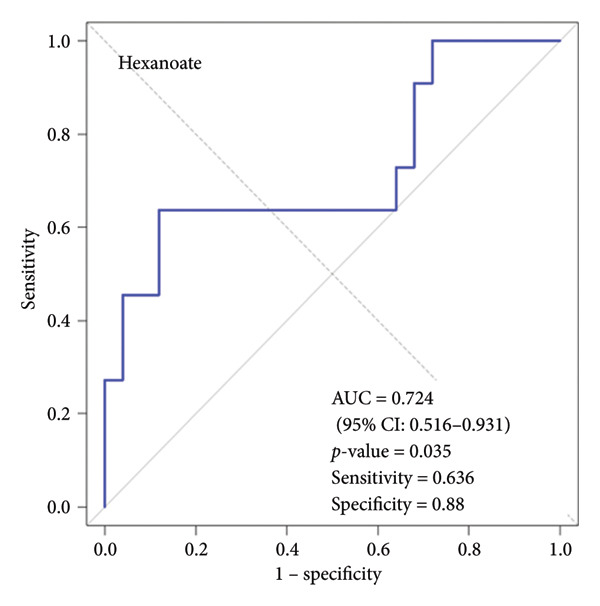
(b)

**Figure:**
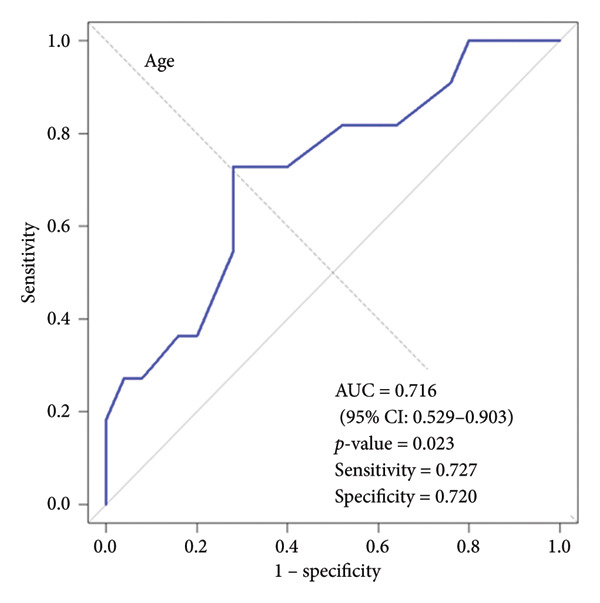
(c)

We then performed univariate logistic regression analysis to identify factors associated with low SMI status. Table 4 presents the results of the univariate logistic regression analysis. Among the variables examined, age was the only factor showing a statistically significant association with low SMI status based on OR (OR: 1.182, 95% CI: 1.024–1.404, *p*‐value = 0.033). The ORs for butyrate (OR: 0.998, 95% CI: 0.993–1.000, *p*‐value = 0.166), hexanoate (OR: 0.976, 95% CI: 0.929–0.999, *p*‐value = 0.210), and other variables did not reach statistical significance in the univariate logistic regression model.

**Table 4:** Univariate and multivariate logistic regression analyses of factors associated with low skeletal muscle index.

Variable	Univariate analysis	*p*‐value	Multivariate analysis	*p*‐value
	Odds ratio (95% CI)		Odds ratio (95% CI)	
Butyrate	0.998 (0.993–1.000)	0.166	NA	NA
Hexanoate	0.976 (0.929–0.999)	0.210	0.980 (0.935–1.002)	0.269
1,3‐Diaminopropane	0.742 (0.385–1.085)	0.223	NA	NA
6‐Phosphogluconate	0.575 (0.234–1.033)	0.142	NA	NA
ASA physical status	3.333 (0.714–16.357)	0.125	NA	NA
Age (years)	1.182 (1.024–1.404)	0.033^∗^	1.155 (0.999–1.375)	0.070
Serum albumin	0.315 (0.048–1.733)	0.194	NA	NA
C‐reactive protein	0.903 (0.210–3.776)	0.888	NA	NA
Lymphocyte count	0.514 (0.131–1.750)	0.303	NA	NA

Abbreviations: ASA, American Society of Anesthesiologist; NA, not available.

^∗^ *p*‐value < 0.05.

Based on the results of the univariate analysis, we included variables with *p*‐value < 0.25 in the multivariate logistic regression model. Using stepwise variable selection with the forward method based on AIC, the final multivariate model included age (OR: 1.155, 95% CI: 0.999–1.375, *p*‐value = 0.070) and hexanoate (OR: 0.980, 95% CI: 0.935–1.002, *p*‐value = 0.269). Although neither variable reached statistical significance at the *p*‐value < 0.05 level in the multivariate model, they were both retained in the final model by the stepwise selection process.

## 4. Discussion

We assessed the relationship between salivary metabolites and SMI in older male patients during the perioperative period. Our univariate analysis identified age as a variable significantly associated with low SMI status based on OR. Additionally, ROC curve analysis revealed that two salivary metabolites, butyrate and hexanoate, along with age, had significant discriminatory ability for distinguishing between high and low SMI with AUC values exceeding 0.7. Patients in the low‐SMI group were older than those in the high‐SMI group, but no significant differences were found in general nutritional indicators (such as serum albumin levels, lymphocyte counts, and CRP) between the two groups. This suggests that the muscle mass differences may not be readily detected using conventional nutritional assessment such as blood tests, but could be identified through salivary metabolite profiles in combination with age assessment.

The significant discriminatory ability of age for differentiating high and low SMI observed in our study is consistent with the established relationship between aging and sarcopenia. Age‐related muscle loss is a well‐documented phenomenon, and our findings reinforce the importance of considering age when assessing muscle mass. The significant OR for age indicates the strong influence of aging on muscle mass decline.

Hexanoate is a six‐carbon short‐chain fatty acid (SCFA) produced by several anaerobic bacteria that inhabit the gut [31]. As previously noted, chronic inflammation appears to play a significant role in the development of sarcopenia [12]. However, while in vitro studies have demonstrated the anti‐inflammatory properties of hexanoate [32–34], whether these effects directly contribute to muscle mass preservation in vivo has not been established. Therefore, further mechanistic studies, including animal models and clinical intervention trials, are needed to clarify the biological pathways linking salivary SCFAs to muscle mass regulation. Meanwhile, in our analysis, hexanoate demonstrated significant discriminatory ability for SMI classification, suggesting its potential utility as a biomarker for muscle mass assessment. In our multivariate analysis using AIC‐based stepwise selection, age and hexanoate were retained in the final model (age: OR 1.155, 95% CI: 0.999–1.375, *p*‐value = 0.070; hexanoate: OR 0.980, 95% CI: 0.935–1.002, *p*‐value = 0.269). While these variables were selected through the model optimization process, neither reached statistical significance (*p*‐value < 0.05). Given the exploratory nature of this study, further validation in larger prospective studies is needed.

Interestingly, although we observed differences in salivary metabolite profiles between high‐ and low‐SMI groups, traditional inflammatory markers such as CRP did not differ significantly between the groups. This discrepancy may reflect that salivary metabolites and serum markers represent different physiological processes. Recent evidence suggests that gut microbiota‐derived SCFAs can influence immune responses in distant organs, including the oral cavity [35], though the underlying mechanisms linking these findings to SMI in our study require further investigation.

Butyrate, another SCFA identified in our analysis, is derived from the intestinal flora and has been suggested to play a role in maintaining muscle mass through energy supply and inflammation control [36]. Butyric acid has also been shown to promote oxidative phosphorylation in the mitochondria of muscle cells [37, 38]. It may inhibit the production of inflammatory cytokines, particularly interleukin‐6, and exert a protective effect against changes in age‐related inflammatory pathways that contribute to a decline in skeletal muscle function and muscle mass [39–41]. In our study, butyrate showed the highest discriminatory ability among the variables examined, indicating its potential as a biomarker associated with skeletal muscle mass. This aligns with previous research indicating that chronic inflammation plays an important role in age‐related muscle loss or sarcopenia [12].

This study suggests the potential of salivary metabolomics as an alternative nutritional assessment method for patients who cannot undergo measurements using conventional BIA devices such as InBody. The SMI differences that were not detected using general nutritional blood indicators could potentially be identified using the salivary metabolic profile. Additionally, this study highlights the potential of noninvasive methods for assessing muscle mass, which is a crucial factor in the perioperative management of the older patients. Older adult patients who are at high risk of postoperative frailty and ICU‐AW require intermittent monitoring of their nutritional status. For this patient cohort, the salivary biomarkers identified in our study could serve as new indicators for monitoring their nutritional status.

This study has several limitations. First, the patients in the low‐SMI group were older than those in the high‐SMI group, thus requiring careful consideration of age in the analysis. However, the lack of differences in general nutritional indicators suggests that salivary metabolites provide additional information beyond age alone. Our multivariate analysis using stepwise variable selection attempted to address this by including age as a covariate. Age and hexanoate were retained in the final model through the selection process, though neither reached statistical significance. Second, this was a retrospective observational study with a relatively small sample size, which may have led to insufficient statistical power to detect weak associations and may have affected the stability of the multivariate model. Therefore, the observed relationships, particularly those involving variables retained by stepwise selection but not reaching statistical significance, should be interpreted with caution. Validation in larger prospective cohorts is warranted to confirm these findings and provide more robust statistical evidence. Third, this study was limited to male patients only. This decision was made for two reasons: first, the SMI cutoff values differ between sexes (7.0 kg/m^2^ for men versus 5.7 kg/m^2^ for women according to the Asian Working Group for Sarcopenia guidelines), requiring sex‐specific analyses; second, the number of female participants who met our inclusion criteria was insufficient to provide adequate statistical power for meaningful analysis. We acknowledge that sex differences in muscle metabolism may exist, and our findings cannot be generalized to female patients. Future studies with larger sample sizes should include both sexes to comprehensively understand the relationship between salivary metabolites and muscle mass across different populations. Fourth, we did not control for potential confounding factors such as diet and gut microbiota composition, which could influence salivary metabolite profiles. Future studies should incorporate dietary assessment and gut microbiota analysis to better understand the relationship between these factors and muscle mass. Fifth, while we observed associations between salivary SCFAs and SMI, the direct mechanism by which gut microbiota metabolites such as hexanoate appear in saliva remains unclear. Recent evidence suggests that SCFAs can enter systemic circulation and influence distant organ systems, including the oral cavity [35], but further mechanistic studies are needed to clarify these pathways in the context of sarcopenia. Sixth, while we observed differences in salivary metabolite profiles between high‐ and low‐SMI groups, traditional serum inflammatory markers (CRP, albumin, lymphocyte count) showed no significant differences. This discrepancy may reflect several possibilities: salivary metabolites may represent local physiological processes distinct from those reflected by serum markers; the influence of sample size, measurement timing, and potential confounding factors cannot be excluded; or gut microbiota‐derived SCFAs may influence oral immunity through systemic circulation [35]. However, these interpretations remain speculative, and experimental validation is required to elucidate the mechanistic basis of these observations.

In the future, larger‐scale prospective studies will be required for verification. Evaluating the prognostic ability through longitudinal measurement during the perioperative period is also important. By clarifying how fluctuations in the metabolite markers identified in this study relate to perioperative complications and patient prognosis, the clinical utility of nutritional assessment using salivary metabolomics will become clearer.

## 5. Strengths and Limitations

Strengths of this study are as follows:

- Noninvasive saliva collection method applicable to patients with contraindications to BIA,
- Use of absolute quantification with standard mixtures for all metabolites,
- Paired measurements obtained within 30 days.

Limitations of this study are as follows:

- Small sample size,
- Retrospective study design preventing causal inference,
- Inclusion of only male patients, limiting generalizability to females,
- Lack of control for potential confounding factors such as diet and gut microbiota composition,
- Single‐center study potentially limiting generalizability.

## 6. Conclusions

In conclusion, this exploratory study suggests that salivary metabolites, particularly butyrate, hexanoate, along with age, may have potential utility for discriminating between high and low SMI in older male patients. However, in our multivariate analysis, although age and hexanoate were retained through AIC‐based stepwise selection, neither reached statistical significance. Despite this limitation, these results suggest the potential utility of salivary metabolomics for the noninvasive assessment of muscle mass status, particularly in patients for whom InBody cannot be used.

**Nomenclature**
AUC: Area under the curve
BIA: Bioelectrical impedance analysis
BMI: Body mass index
BMR: Basal metabolic rate
CRP: C‐reactive protein
ICU‐AW: Intensive care unit–acquired weakness
PCA: Principal component analysis
ROC: Receiver operating characteristic
SCFA: Short‐chain fatty acids
SMI: Skeletal muscle index

## Ethics Statement

This study was conducted with the approval of the Ethical Review Committee of Yamagata University Faculty of Medicine (approval number: 2024–60).

## Consent

The study details were published on the Yamagata University website, and participants were provided with an opt‐out document to ensure that they had the opportunity to express their intention to refuse participation. Written consent was obtained where applicable, and all personal information was anonymized.

Written consent for publication was not obtained from the participants, but the opt‐out method was used.

## Disclosure

All authors have read and approved the final version of the manuscript.

## Conflicts of Interest

The authors declare no conflicts of interest.

## Author Contributions

Tatsuya Hayasaka and Shigeo Ishikawa contributed to study design. Data collection and analysis were performed by Tatsuya Hayasaka, Shigeo Ishikawa, Ayuka Narisawa, and Machika Moriya. Tatsuya Hayasaka, Shigeo Ishikawa, Hiroaki Toyama, and Masahiro Sugimoto carried out manuscript preparation and revision.

## Funding

The authors received no specific funding for this work.

## Supporting Information

Supporting Table provides the detailed results of ROC curve analysis, including optimal cut‐off values determined by the Youden index, sensitivity, specificity, Youden index values, AUC with 95% confidence intervals, and *p*‐value for butyrate, hexanoate, and age in discriminating between high SMI (≥ 7.0 kg/m^2^) and low SMI (< 7.0 kg/m^2^) groups.

## Supporting information


**Supporting Information** Additional supporting information can be found online in the Supporting Information section.

## Data Availability

Data are available on request due to privacy/ethical restrictions.
